# Key factors behind various specific phobia subtypes

**DOI:** 10.1038/s41598-023-49691-0

**Published:** 2023-12-14

**Authors:** Andras N. Zsido, Botond L. Kiss, Julia Basler, Bela Birkas, Carlos M. Coelho

**Affiliations:** 1 Institute of Psychology, University of Pécs, 6 Ifjusag Street, Pécs, Baranya 7624 Hungary; 2 Szentágothai Research Centre, University of Pécs, Pécs, Hungary; 3 Medical School, University of Pécs, Pécs, Hungary; 4 Department of Psychology, University of the Azores, Ponta Delgada, Portugal

**Keywords:** Anxiety, Risk factors

## Abstract

While it has been suggested that more than a quarter of the whole population is at risk of developing some form of specific phobia (SP) during their lives, we still know little about the various risk and protective factors and underlying mechanisms. Moreover, although SPs are distinct mental disorder categories, most studies do not distinguish between them, or stress their differences. Thus, our study was manifold. We examined the psychometric properties of the Specific Phobia Questionnaire (SPQ) and assessed whether it can be used for screening in the general population in a large sample (N = 685). Then, using general linear modeling on a second sample (N = 432), we tested how potential socio-demographic, cognitive emotion regulatory, and personality variables were associated with the five SP subtypes. Our results show that the SPQ is a reliable screening tool. More importantly, we identified transdiagnostic (e.g., younger age, female gender, rumination, catastrophizing, positive refocusing) as well as phobia-specific factors that may contribute to the development and maintenance of SPs. Our results support previous claims that phobias are more different than previously thought, and, consequently, should be separately studied, instead of collapsing into one category. Our findings could be pertinent for both prevention and intervention strategies.

## Introduction

Evidence shows that specific phobias (SPs) are the most common anxiety- and mental disorders with a lifetime prevalence between 7.4 and 14% among adults with a cumulative incidence of 27% that is increasing^1–3^. That is, over a quarter of the whole population is at risk of developing some form of SP throughout their lives. Fear and its automatic activation by the detection of an object that might signal danger^4^ is an adaptive response to imminent threats insofar as it helps prepare the organism for action and reduce the risk of being harmed. However, excessive levels of fear can interfere with one’s cognitive processes and movement preparation, and, as a consequence may result in disrupted behavior^5^; for example, a diver might ascend too fast, a pedestrian might freeze, or a policeman might freeze or shoot too early^6^. Such core negative experiences—accompanied by a panic-like fear response and a loss of control over one’s emotions and behaviors—can result in the development of SPs^7^. Even in the absence of a proportional danger, phobias then manifest as extreme fear, and can be triggered even by the thought of the feared object^8,9^. The possibility and likelihood of direct engagement with potential treats (e.g., spiders, snakes, storms, etc.) may affect the prevalence of^10,11^ Thus, environmental conditions have an influence on the development of SPs. The 5th edition of the Diagnostic and Statistical Manual for Mental Disorders (DSM-5) distinguishes five SP subtypes: animals (e.g., snake, spider), environmental (e.g., storm, heights), situational (e.g., enclosed spaces), blood-injection-injury (BII; e.g., medical examinations), and other (e.g., choking). This categorization can also be useful in understanding fears that are not yet excessive but may foreshadow the possibility of developing a phobia (i.e., subclinical SP).

Determining the percentage of the general population that may be affected by subclinical levels of fear is vital, as SPs are often unrecognized and, consequently, often go untreated for a long time^12^. A lack of screening, diagnosis, and treatment has negative consequences both at an individual (e.g., reduced quality of life) and at a societal level (e.g., economic costs)^13–15^. A recent study offers a quick screening tool to assess the five subtypes of SP, the Specific Phobia Questionnaire (SPQ)^16^. The SPQ measures both fear and the extent to which fear interferes with daily life and has been proven reliable in clinical and subclinical samples as well. Assessing both fear and daily life interference is a novelty of the questionnaire and is in line with DSM-5 requirements for phobia diagnosis^8,17,18^. The tool is capable of identifying those at risk of either SP subtypes. Since SPQ has only been published in recent years, further evidence is needed about its psychometric soundness. While previous studies warn that a considerable percentage of the whole population might be affected by SPs at some point in their lives^3^, we still do not know the exact number and how it varies across countries^2^. The use of SPQ also opens the possibility of closely monitoring the percentage of the population at risk of various SPs.

While a large proportion of the population is at risk of developing SP, to date, still little is known about the particular risk factors associated with the development. The risk factors previous studies have shown can be categorized into three large groups: socio-demographic, personality, and cognitive emotion regulation (ER) strategies. A large-scale investigation in a representative sample of community-dwelling adults^19^ has shown that the most prominent risk factors were female sex, a comorbid diagnosis of lifetime major depressive disorder, having experienced traumatic experiences involving significant others, the number of chronic diseases, and a comorbid diagnosis of substance use. Other studies also point to these factors, as well as higher levels of depressive mood and fewer years of education as potential risks of developing a SP^1,20,21^. Similarly, powerlessness, loss of control, and the lack of perceived control (strongly linked to one’s desire for control) and, consequently, the excessiveness of worry have long been associated with SPs^22–24^. It has also been shown^25–27^ that SPs are associated with emotion dysregulation problems (e.g., using putatively maladaptive emotion regulation strategies, such as rumination). Yet, past studies have not sought to answer the question of whether these risk factors are transdiagnostic for all SPs or whether there is a specific pattern unique to each subtype.

The factors that increase the likelihood of reaching an excessive level of fear, and potentially the development of phobia, might vary across SP subtypes. There is great variability in the prevalence of SP subtypes which might indicate that besides the universal risk factors, there are subtype-specific ones as well. The key stimulus element that triggers the fear response greatly varies across SPs^28^. Consequently, there are overlaps but also unique characteristics of each SP in terms of the connected risk factors^28^. In fact, as part of the Netherlands Mental Health Survey and Incidence Study, it has been shown^17^ that the likelihood of impairment, comorbidity, and personality problems also greatly vary across SP subtypes. Similarly, there is evidence that a phobia-specific pattern exists in the putatively adaptive and maladaptive cognitive ER strategies^25^. Further, neurological evidence shows that viewing various phobia-relevant objects results in a different activation pattern^23,29–33^, which also points to the direction that SP subtypes might be more different than previously thought. These results together underscore that there might be differences in the socio-demographic, cognitive emotion regulatory, and personality factors behind various SPs. Mapping the underlying transdiagnostic and unique factors for each SP can be crucial for an effective intervention therapy and may also increase the efficiency of prevention to deal with SPs.

The overarching goal of this study was twofold. First, we sought to test the SPQ in a large sample of community-dwelling adults, describe the prevalence of SP subtypes, and help establish standard scores. Second, we wanted to explore the unique and transdiagnostic risk and protective factors across SP subtypes associated with the level of fear and the interference this fear causes. We hypothesized that some factors, such as younger age, female gender, more previous traumatic life events, depressive mood, emotion dysregulation, and worry will be associated with higher rates of fear and interference. However, we also predicted that each SP subtype will have a unique pattern of associated risk factors concerning the cognitive ER strategies involved and other, perceived control-related components. To our knowledge, to this date, this is the only study to separately investigate risk and protective factors in various SP subtypes. Our results may assist counselors, social workers, and other health professionals in identifying individuals who might be at risk of developing an SP. Applications of these findings are pertinent for both prevention and intervention strategies.

## Methods

### Participants

We used two separate samples in this study. The first sample was used to test the psychometric properties of the SPQ and for descriptive analysis to estimate the prevalence of the five SP subtypes. For the first sample, we recruited 685 Caucasian participants (447 females), aged 18–85 years (M = 29.1, SD = 12.8). Here, we wanted to reach a large number of respondents to have a large enough sample for the descriptive analysis. For statistical purposes, we intended to increase the number of respondents by limiting the test battery to questions regarding age, gender, and the SPQ.

Then, we sought to test which sociodemographic, life history, cognitive emotion regulation, and personality-related factors play a key role in the development and maintenance of fears related to different SP subtypes. The second sample comprised 432 Caucasian participants (347 females), aged 18–67 years (M = 26.5, SD = 9.46). Here, the required sample size was determined by computing estimated statistical power with a conservative approach (f^2^ = 0.10, β > 0.95, alpha = 0.05) using G*Power 3 software^34^. The analysis indicated a required minimum sample size of 373; thus, our study was adequately powered. Table 1 shows the central tendencies of the questionnaires and more details about the sample.

**Table 1:** Detailed descriptive statistics of the sample including demographic variables and the questionnaires used in the study separately for the first (N = 685) and the second (N = 432) sample.

		Male	Female	Other		
Subsample 1 (N = 685, missing = 11)						
Gender	Count	238	447	0		
	%	34.7	65.3	0		
Subsample 2 (N = 429, missing = 3)						
Gender	Count	79	344	6		
	%	18.4	80.2	1.4		

		Single	In a relationship	Married	Divorced	Widow(er)
Marital status	Count	174	168	81	8	1
	%	40.3	38.9	18.8	1.9	0.2

		Primary school	High school	In higher education	BA/MA	PhD
Education	Count	171	168	81	8	1
	%	39.9	39.2	18.9	1.9	0.2

		Never	Rarely	Sometimes	Frequently	
Smoking	Count	226	67	69	67	
	%	52.7	15.6	16.1	15.6	

		Never	Rarely	Sometimes	Frequently	
Marihuana	Count	341	63	13	12	
	%	79.5	14.7	3.0	2.8	

		No	Yes, but not in the last 12 months	Yes, also in the last 12 months		
Addiction diagnosis	Count	423	1	5		
	%	98.6	0.2	1.2		

		No	Yes, but not in the last 12 months	Yes, also in the last 12 months		
Depression diagnosis	Count	372	18	39		
	%	86.7	4.2	9.1		

		No	Yes			
Phobia diagnosis	Count	417	12			
	%	97.2%	2.8%			

		M	SD			
Chronic diseases	No. of	0.284	0.781			
Traumatic experiences	No. of	3.64	3.70			
Violent experiences	No. of	0.822	2.40			
Traumatic experiences of close others	No. of	1.48	3.07			
CERQ	SB	5.48	2.13			
	ACC	6.69	1.96			
	RUM	6.89	2.23			
	REF	4.53	2.11			
	PLAN	7.05	1.82			
	REAP	6.67	2.12			
	PP	6.14	2.18			
	CAT	4.62	2.06			
	OB	3.71	1.40			
DSC	Total	93.2	15.0			
ACQ	EC	14.7	4.50			
	TC	21.9	4.45			
	SC	12.8	3.37			
BDI	Total	4.34	4.89			
PWSQ	Total	17.3	3.23			

*CERQ* cognitive emotion regulation scale, *DSC* desirability of control scale, *ACQ* anxiety control questionnaire, *BDI* beck depression inventory, *PWSQ* Penn state worry questionnaire.

All participants were recruited through the Internet by posting invitations on various forums and mailing lists to obtain a non-clinical heterogeneous sample. Our goal was to obtain a heterogeneous sample representing people from a variety of demographic, socio-economic, and educational backgrounds. Table 1 shows the central tendencies of the questionnaires and more details about the samples. None of the respondents reported having been diagnosed with a specific phobia by a clinician or psychiatrist. Subjects participated voluntarily. The research was approved by the Hungarian United Ethical Review Committee for Research in Psychology and was carried out in accordance with the Code of Ethics of the World Medical Association (Declaration of Helsinki). Informed and written consent was obtained from all participants.

### Questionnaires

#### Socio-demographic questions

Socio-demographic questions were based on the results of a previous large-scale representative study on specific phobias^19^ and included age, gender, marital status, the highest level of education, the number of personally experienced traumatic and violent life experiences, witnessed traumatic life experiences, the number of chronic diseases, smoker status, alcohol, marijuana, and other substance consumption habits, diagnosis of substance abuse disorder and major depressive disorder. These questions were selected because they emerged as significant predictors of phobias.

#### Specific phobia questionnaire (SPQ)

The SPQ measures the five subtypes of specific phobias with 43 items^16^. Each item is evaluated on two 5-point Likert-type scales (0—None to 4—Extreme) Fear and Interference. The Fear scales measure how fearful the respondent is of each situation, while the Interference scales measure how much the respondent’s fear interferes with their lives. The McDonald’s omegas for the Fear and Interference scales (respectively) were 0.78 and 0.84 (animals), 0.77 and 0.82 (natural environment), 0.77 and 0.78 (situational), 0.92 and 0.93 (blood-injection-injury). The Spearman–Brown coefficients for the other subscale were 0.50 and 0.65. The reason behind the lower reliability value of the other subscale is presumably due to the fact that it only consists of two items (Pearson r = 0.33 and 0.49).

All of the participants filled out the Hungarian language versions of the scales. The process of translation and adaptation of the instruments followed the recommendations of the American Psychiatric Association^8^. First, the original version of the questionnaire was given to two psychologists, both of whom were fluent in English, to translate the SPQ into Hungarian. Then, a third person, an expert in test development, was asked to compare the two versions and merge them into one to avoid any discrepancies and mistranslations. Subsequently, a person with a Master’s degree in psychology who is fluent in English translated this version back into English. Thereafter, an expert panel consisting of researchers in psychology as well as a native English speaker reviewed the back-translated version. They revised and corrected the Hungarian version to make it as close as possible in meaning to the original SPQ. We did not change any aspect of the original scale.

#### Cognitive emotion regulation questionnaire (CERQ)

The 18-item version of the CERQ^35,36^ measures cognitive strategies that characterize the individual’s style of responding to stressful events. The questionnaire has 9 subscales in total, four subscales measure putatively maladaptive strategies (Self-blame, Rumination, Catastrophizing, and Other blame), and five measure putatively adaptive strategies (Acceptance, Positive refocusing, Refocus on planning, Positive reappraisal, and Putting into perspective). Items are measured on 5-point Likert scales (1—almost never to 5—almost always). Higher scores indicate that a person uses the given strategy more often. The McDonald’s omegas were 0.77 (self-blame), 0.86 (rumination), 0.81 (catastrophizing), 0.63 (other blame), 0.84 (acceptance), 0.85 (positive refocusing), 0.63 (refocus on planning), 0.74 (positive reappraisal), and 0.79 (putting into perspective).

#### Desirability of control

To measure the level of motivation to control the events in one's life we used the Desirability of Control questionnaire (DSC)^37^. The questionnaire is a one-scale tool comprising 20 items. Items are rated on 7-point Likert-type scales (1—doesn’t apply to me to 7—always applies to me). Higher scores indicate a stronger desirability of control. The McDonald’s omega was 0.83.

#### Perceived emotional control

The short, 15-item version of the Anxiety Control Questionnaire (ACQ) was used to assess three facets of perceived control: Emotion Control, Threat Control, and Stress Control^38^. Items are rated on 6-point Likert-type scales (0—strongly disagree to 5—strongly agree). Higher scores indicate a higher level of perceived control. The McDonald’s omegas were 0.79 (emotion control), 0.76 (threat control), and 0.72 (stress control).

#### Depression

The 6-item short version of the Beck Depression Inventory (BDI) was used to measure depressive mood^39^. Items were presented on 4-point scales, similarly to the original 21-item version. Higher scores suggest increased depressive symptomatology. The McDonald’s omega was 0.77.

#### Worry

The brief, 5-item version of the Penn State Worry Questionnaire (PWSQ) was used to measure the tendency, intensity, and uncontrollability of worry^40^. Items are rated on 5-point Likert-type scales (1—not at all typical of me to 5—very typical of me). Higher scores indicate a higher propensity to worry. The McDonald’s omega was 0.84.

### Statistical analyses

There were no missing data because the answer was made mandatory for each question in the online surveys. We did not find any indicators of bot responses, and we did not expect to see any because participants completed all surveys voluntarily and in no instance were given any compensation. We sought for outliers who were ± 3 SDs away from the mean but we found none (which is justified by the large sample size). We also sought duplicate responses and identified four in the first sample; these were removed before the data analysis. We used Jamovi statistical software version 2.3^41^ for the data analysis.

Before addressing the first objective of the study, we wanted to demonstrate that the factor structure of the SPQ suggested by the original authors is valid on an independent sample. Thus, we started by testing the five-factor model (separately for fear and interference) suggested by previous studies with confirmatory factor analysis on the first sample. We used the diagonally weighted least squares (DWLS) estimator. To assess model fit, we used the comparative fit index (CFI), the Tucker–Lewis index (TLI), the Parsimony Normed Fit Index (PNFI), the root mean square error of approximation (RMSEA), and the standardized root mean squared residual index (SRMR). The cutoffs for good model fit were CFI and TLI values of 0.95 or greater^42^, PNFI value of 0.8 or greater^43^, RMSEA and SRMR values of 0.08 or lower^44^. McDonald’s omega values were also calculated to assess the reliability of the scales.

Then moving on to the first objective of the study, using the first sample, we then examined our sample with respect to the cut-off scores suggested by the developers of SPQ^16^ to report the prevalence of each SP subtype. After this, gender differences were examined using a pairwise comparison with Student’s t-test, and the possible effects of age were investigated using Pearson correlation analysis. Where normality was violated (i.e., he absolute values of Skewness and Kurtosis were greater than 2), robust alternatives (i.e., Mann–Whitney test and Spearman correlation) were used. Regarding the comparison of effect sizes between parametric and nonparametric tests the guidelines by Cohen may serve as a good basis^45^. Pearson and Spearman correlations may be interpreted along the same guidelines, i.e., an r value between 0.2 and 0.5 refers to medium effect size. For Cohen’s d (Student t test) the range of medium effect is 0.5–0.8., while for the rank biserial correlation (Mann–Whitney test) the medium effect size is 0.4–0.8. Values below can be considered small effect sizes and values above can be considered large effect sizes.

Then to address our second objective, on the second sample, we used General Linear Modelling (GLM) to explore the socio-demographic factors, cognitive emotion regulation strategies, and personality-related questionnaires that are significant predictors of SPQ subscale scores. We used the five Fear and five Interference SPQ scores as the dependent variables in separate models. For each dependent variable, we tested three models. In *Model 1* we tested the effects of socio-demographic variables, therefore the independent predictors were age, gender, marital status, and the level of education, the predictors were the number of personally experienced traumatic and violent life experiences, witnessed traumatic life experiences, the number of chronic diseases, smoker status, alcohol, marijuana, and other substance consumption habits, diagnosis of substance abuse disorder and major depressive disorder. In *Model 2* we tested the effects of cognitive ER strategies, thus the predictors were the nine CERQ subscales. Finally, in *Model 3* we tested personality-related factors, so the predictors were the DSC, three ACQ subscales, BDI, and PWSQ scores. The assumption of normality was not violated. The absolute values of Skewness and Kurtosis were less than 2 for all SPQ Fear subscales. We used Box-Cox transformation (lambda = 0.5) on the Interference subscales to achieve normal distribution. Statistical results will be presented in tables instead of in the text to make the description of the results easier to follow and more understandable.

### Ethical approval

Ethics approval was obtained from the Hungarian United Ethical Review Committee for Research in Psychology.

### Informed consent

Informed consent was obtained from all individual participants included in the study.

## Results

### Factor structure and descriptive analysis

**Figure 1:**
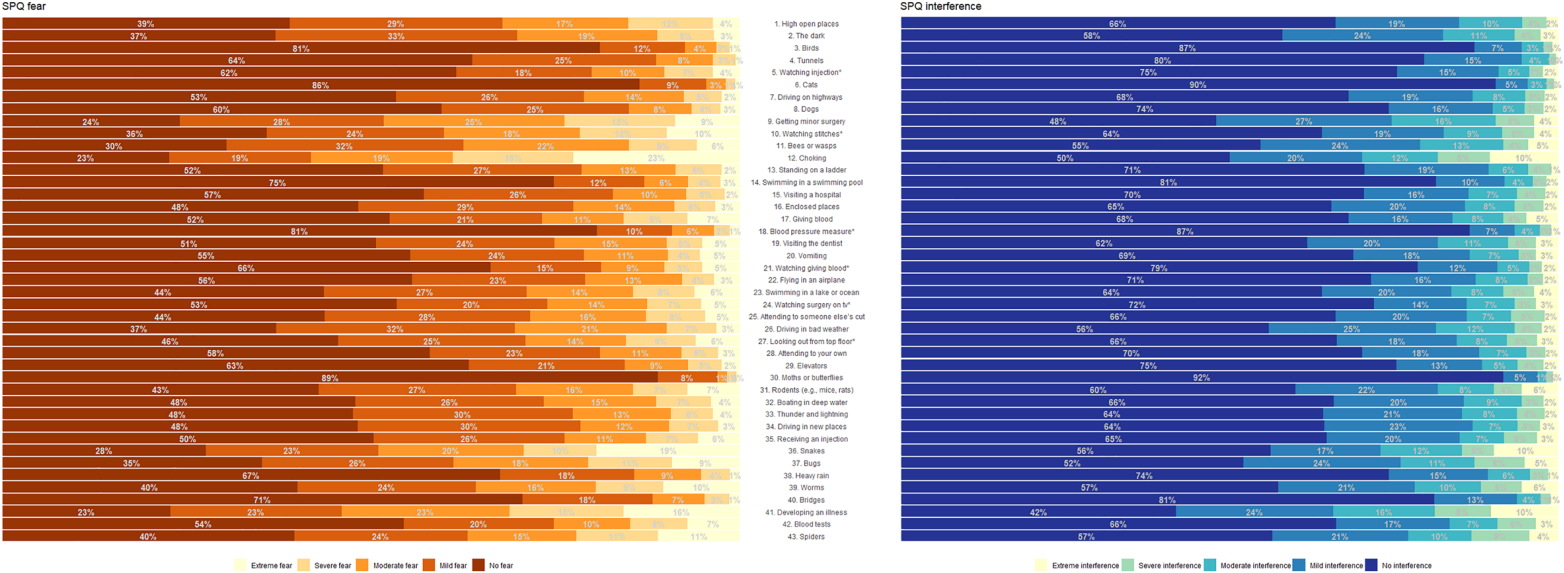
Percentage distribution of responses (no, mild, moderate, severe, extreme fear/interference) on the two subscales (Fear and Interference) of the Specific Phobia Questionnaire (SPQ). The left panel with the brown-yellow scale indicates the distribution of responses to the Fear subscale, where brown is no fear and yellow is extreme fear. The right panel with the blue-yellow scale indicates the distribution of responses to the Interference subscale, where blue is no fear and yellow is extreme fear. Corresponding items of the questionnaire are displayed in the middle.

The two, five-factor structure of the SPQ showed acceptable fit for the Fear (X^2^ (830) = 4366.216, p < 0.001, CFI = 0.991 TLI = 0.991, PNFI = 0.935, RMSEA = 0.062 [90% CI: 0.060–0.064], SRMR = 0.052) and the Interference subscales (X^2^(850) = 6566.073, p < 0.001, CFI = 0.994 TLI = 0.994, PNFI = 0.909, RMSEA = 0.078 [90% CI: 0.076–0.080], SRMR = 0.048). Factor loadings for the Fear scale ranged between 0.623 and 0.835 and between 0.734 and 0.906 for the Interference scale. Figure 1 shows the distribution of answers on the sample.

The score corresponding to the 95th percentile on a given questionnaire is often considered a clinically significant limit^46–49^. Table 2 shows the 95th percentile scores separately for subscales and total score on the Fear and Interference scales as well as the number and percentage of participants who reached this score separately for males and females.

**Table 2:** The questionnaire scores for subscales and total scores of the specific phobia separately for the fear (F) and interference (I) scales and corresponding prevalence values based on the 95th percentile and cut-off points suggested by the authors of the original questionnaire separately for males, females, and the total sample.

Subscale	Cut-off	Number			Percentage		
		Total	Male	Female	Total	Male	Female
95th percentile scores							
Animals (F)	19	43	6	37	6.277	2.521	8.277
Animals (I)	19	40	8	32	5.839	3.361	7.159
Environment (F)	20	39	6	33	5.693	2,12	7.383
Environment (I)	17	40	9	31	5.839	3.782	6.935
Situation (F)	16	36	8	28	5.255	3.361	6.264
Situation (I)	14	38	11	27	5.547	4.622	6.040
BII (F)	36	31	4	27	4.526	1.681	6.040
BII (I)	30	36	8	28	5.255	3.361	6.264
Other (F)	7	43	4	39	6.277	1.681	8.725
Other (I)	6	39	5	34	5.693	2.101	7.606
Total (F)	87	34	5	29	4.963	2.101	6.488
Total (I)	77	36	7	29	5.255	1.566	6.488
Based on cut-off points							
Animals (F)	8	335	68	267	48.905	28.571	59.732
Animals (I)	8	210	46	164	30.657	19.328	36.689
Environment (F)	15	105	17	88	15.328	7.143	19.687
Environment (I)	15	58	11	47	8.467	4.622	10.515
Situation (F)	4	413	97	316	60.292	40.756	70.694
Situation (I)	4	258	58	200	37.664	24.370	44.743
BII (F)	20	166	38	128	24.234	15.966	28.635
BII (I)	20	79	18	61	11.533	7.563	13.647
Other (F)	6	84	8	76	12.263	3.361	17.002
Other (I)	6	39	5	34	5.693	2.101	7.606

Values presented refer to the number and percentage of participants who reach the corresponding cut-off scores.

*BII* blood-injection-injury.

Further, based on the cut-off point suggested by the authors of the original questionnaire a large portion of the respondents could be considered at risk of having an SP. The prevalence values range from 12 to 60%. The exact cut-off values for the Fear and Interference subscales and the number and percentage of participants at risk of SP are shown in Table 2.

Further analysis revealed systematic gender differences: females scored higher than males on all subscales both on the Fear scale and the Interference scale. Detailed statistical results are displayed in Table 3. Regarding the relationship between SPQ and age, the Spearman correlation (controlled for gender differences) revealed significant but weak positive correlations for all the Interference scores (range 0.09–0.15), while we found no significant results for the Fear scale. The exact correlational values are displayed in Table 3.

**Table 3:** Descriptive statistics (mean score and standard deviation) for subscales and total scores of the Specific Phobia separately for the Fear (F) and Interference (I) scales.

Subscale	Total, mean (SD)	Age correlation	Age correlation (controlling for gender)	Male, mean (SD)	Female, mean (SD)	Gender difference
Total (F)	39.6 (24.5)	Rho = − 0.041, p = 0.287	Rho = − 0.012, p = 0.759	28.6 (21.3)	45.4 (24.1)	t(522)* = − 9.28; p < 0.001; Cohen’s d = − 0.737
Total (I)	24.6 (24.7)	Rho = 0.117, p = 0.002	Rho = 0.139, < 0.001	18.3 (21.7)	28.0 (25.5)	U = 39,109; p < 0.001; r = 0.265
Animals (F)	8.21 (5.98)	Rho = − 0.072, p = 0.060	Rho = − 0.046, p = 0.228	5.79 (5.52)	9.50 (5.82)	t(507)* = − 8.21, p < 0.001, Cohen’s d = − 0.654
Animals (I)	5.95 (6.50)	Rho = 0.073, p = 0.057	Rho = 0.092, p = 0.016	4.29 (5.36)	6.84 (6.87)	t(593)* = − 5.37; p < 0.001; Cohen’s d = 0.414
Environment (F)	7.94 (6.25)	Rho = 0.039, p = 0.304	Rho = 0.069, p = 0.071	5.53 (5.17)	9.22 (6.40)	t(577)* = − 8.16, p < 0.001, Cohen’s d = − 0.634
Environment (I)	4.82 (5.84)	Rho = 0.130, p < 0.001	Rho = 0.148, < 0.001	3.49 (4.85)	5.53 (6.19)	U = 41,598; p < 0.001; r = 0.218
Situation (F)	5.93 (4.96)	Rho = 0.019, p = 0.614	Rho = 0.047, p = 0.218	4.12 (4.31)	6.90 (5.01)	t(549)* = − 7.58; p < 0.001; Cohen’s d = − 0.594
Situation (I)	3.85 (4.83)	Rho = 0.142; p < 0.001	Rho = 0.163, < 0.001	2.82 (4.42)	4.41 (4.94)	U = 40,510; p < 0.001; r = 0.238
BII (F)	13.5 (10.9)	Rho = − 0.049, p = 0.198	Rho = − 0.032, p = 0.405	10.3 (9.09)	15.1 (11.4)	t(582)* = − 6.04; p < 0.001; Cohen’s d = − 0.468
BII (I)	8.36 (9.99)	Rho = 0.099; p = 0.010	Rho = 0.111, p = 0.004	6.69 (8.76)	9.24 (10.5)	U = 44,905; p < 0.001; r = 0.156
Other (F)	2.78 (2.15)	Rho = 0.004, p = 0.912	Rho = 0.029, p = 0.443	1.96 (1.83)	3.21 (2.18)	t(562)* = − 7.94; p < 0.001; Cohen’s d = − 0.620
Other (I)	1.62 (1.98)	Rho = 0.119; p = 0.002	Rho = 0.146, < 0.001	0.979 (1.58)	1.96 (2.09)	t(604)* = − 6.86; p < 0.001; Cohen’s d = − 0.528

The statistics regarding correlations with age (with and without controlling for gender) and gender differences are also displayed.

For Mann–Whitney test regarding gender differences, *r* values stand for rank biserial correlation.

*BII* blood-injection-injury.

*Means a Welch-corrected t-test.

### General linear models

#### Fear

We began by examining which socio-demographic factors, ER strategies, and personality-related questionnaires predict the five SPQ subscale scores regarding fear levels. The negative predictors may be considered protective factors because they are associated with lower levels of fear, in contrast, positive predictors can be considered risk factors as these variables are associated with higher levels of fear. Detailed statistical results, including model fit, and individual variable effects are presented in Supplementary material S1 regarding the five Fear subscales of SPQ.

For the *Animals subtype*, we found that age and perceived higher threat control were negative predictors, while female gender, depression diagnosis, a higher number of chronic diseases, using rumination, positive refocusing, and blaming others as ER strategies more often, and higher levels of worrying were all positive predictors.

Regarding the *Environmental* subtype, the significant negative predictors were more frequent alcohol consumption, using refocus on planning to regulate emotions, and perceived higher threat control; positive predictors were using rumination, positive refocusing, catastrophizing as ER strategies, higher BDI score, and worrying.

For the *Situational* subtype, we found that fear was negatively predicted by age and more frequent alcohol consumption, while it was positively predicted by female gender, more traumatic experiences, ruminating and using positive refocusing more often, and worrying.

For the *BII* subtype, the significant negative predictors were age and more frequent marijuana consumption, while positive predictors were female gender, ruminating and catastrophizing more often, and using positive refocusing to regulate emotions.

Finally, regarding the *other* subtype, scores were negatively associated with age, higher levels of education, more frequent alcohol and marijuana consumption, and higher levels of perceived stress control. Scores were positively associated with the female gender, ruminating and catastrophizing more often, and worrying.

In sum, it appears that some factors, such as age, gender, alcohol or marijuana consumption, a tendency to ruminate, use positive refocusing, and worry emerge as transdiagnostic factors appearing as significant predictors in nearly all subtypes. However, each subtype has a unique pattern that includes other significant predictors as well, namely depression diagnosis, chronic diseases and blaming others in animal phobias, refocus on planning and depressive mood in environmental phobias, traumatic experience in situational phobias, level of education and stress control in the other subtype.

#### Interference

We, then also investigated the effects of perceived interference of fears with the socio-demographic factors, ER strategies, and personality-related questionnaires as predictor variables. Again, negative predictors can be considered protective factors because they are associated with lower levels of interference; while positive predictors may be considered risk factors as they are associated with higher levels of interference. Supplementary material S2 shows the detailed statistical results, including model fit, and individual variable effects regarding the five Interference subscales of SPQ.

Regarding the *Animal* subtype, we found that education and using refocus on planning to regulate emotions emerged as significant negative predictors, while the female gender, using rumination, positive refocusing, and catastrophizing as ER strategies were positive predictors.

Regarding the *Environmental* subtype, significant negative predictors were education, more frequent alcohol consumption, using refocus on planning to regulate emotions, and higher perceived threat control. Positive predictors were using self-blame, positive refocusing, catastrophizing ER strategies, and depressive mood.

For the *Situational* subtype, we found that the interference was negatively predicted by education, more frequent alcohol consumption, and using acceptance to regulate emotions; while it was positively predicted by the female gender, higher number of chronic diseases, ruminating and catastrophizing more often and using putting into perspective to regulate emotions, depressive mood, and worrying.

For the *BII* subtype, the significant negative predictors were more frequent marijuana consumption, the use of acceptance, and refocus on planning ER strategies; while positive predictors were ruminating and catastrophizing and using refocus on planning and putting into perspective to regulate emotions.

Finally, regarding the *other* subtype, education, the use of acceptance to regulate emotions, and higher perceived stress control were identified as significant negative predictors, while the number of chronic diseases, catastrophizing, and worrying were positive predictors.

To sum up, we, again found some factors that emerged as transdiagnostic factors across all SP subtypes (e.g., education, a tendency to catastrophize the event); but also found several factors that seem to be subtype-specific. For instance, female gender in animal and situational phobia, self-blame and threat control in environmental phobia, marijuana consumption in BII phobia, and stress control in the other subtype.

## Discussion

Although SP is the most common mental disorder with a 7.4–14% lifetime prevalence and a cumulative incidence of 27%^1–3^, it often goes undiagnosed and untreated for a long time, possibly due to the lack of an appropriate screening tool^12^. The SPQ^16^ offers a quick and reliable way to screen fears and associated interference on the five subtypes of SP; and is capable of identifying those at risk of either SP subtype. Therefore, the first goal of the present study was to examine the reliability of the SPQ in a large sample of community-dwelling adults and describe the prevalence of SP subtypes. Our results have shown that the questionnaire has sound psychometric properties and can be used in a different culture than it was developed. The results and prevalence values are similar to those of the original study. Compared to past studies^1,2^ that used diagnostic interviews and trained personnel for data collection, the number of phobic individuals in our sample differs significantly when the cut-off points suggested by the authors are applied. In our sample, the 5 subtypes (based on fear score) varied between 12.3 and 60.3%. Here, animal phobia had a prevalence of 48.9%, compared to 3.8% in the study by Wardenaar et al. (2017). There was also a large difference regarding Situational (60.3% vs. 6.3%), BII (24.2% vs. 3.0%), and Environmental (15.3% vs. 2.3%) phobias between our and Wardenaar et al.’s study. The large differences may be partially due to the fact that the SPQ questionnaire, based on the cut-off points suggested, is not a diagnostic tool but rather a screening tool for the early identification of those at risk of SP. A higher score in this case indicates more susceptibility to these types of fears. In this way, the questionnaire can help to identify the groups most at risk of such fears, who can then be interviewed by a clinician and given appropriate help. A possible solution could be using the 95th percentile scores demonstrated in the present study as cut-offs in future research. In sum, our results provide further evidence that screening for SPs is important as it may help identify people at an early, subclinical stage where prevention can be more successful and easier than after the development of a disorder. Moreover, we have provided further evidence that SPQ is a reliable screening tool.

We sought to explore the transdiagnostic risk and protective factors across SP subtypes associated with the level of fear and the interference this fear causes as still little is known about the socio-demographic, cognitive emotion regulatory, and personality risk factors related to the development of these disorders. As expected, the factors associated with higher fear were younger age, female gender, rumination, positive refocusing, and worrying; while female gender, fewer years of education, and catastrophizing were associated with interference. Regarding gender and age, we found that females scored higher than males across all SP subtypes and had a weak negative correlation with age. These results are in line with previous studies showing a similar gender and age effect^2,50–52^ as well as the fact that females compared to males are more likely to be diagnosed with SP^53,54^. This is also in line with the results of past studies^1,16,20,21,23,25,55^ and suggests the notion that there are shared factors across SP subtypes. The fact that the prevalence and intensity of most phobias tend to decrease with age^56^ and that females are at higher risk of developing an SP is well-established^57^. Similarly, emotion dysregulation is strongly associated with SPs and anxiety disorders as they can augment fears^58–60^. Focusing on negative emotions and failing to appropriately regulate emotions can increase worrying^61,62^, enhancing symptoms of anxiety^63^, therefore, augmenting everyday life interference. Our result suggests that positive refocusing, a putatively adaptive ER strategy also augments fears. Although an ER strategy may not inherently be either adaptive or maladaptive, this association may still seem contradictory. However, recent evidence^64,65^ showed that adaptive ER strategies, if not used properly, may be associated with lower well-being and life satisfaction. Using positive refocusing means that the person thinks about positive, happy, and pleasant experiences instead of about current negative events^66^. A possible explanation behind our result is that positive refocusing might appear as an avoidance strategy in the case of phobias. Avoidance, in any form, is not an adaptive behavior insofar as it enhances fear^67^ and was associated with psychopathology^65^.

We also wanted to investigate the unique pattern of risk factors associated with SPs, as there is great variability in prevalence^2^, stimulus element triggering fear^28^, the likelihood of impairment, comorbidity, and personality problems^17^, cognitive ER strategies^25^ and brain activation pattern^23,29,30^ of SP subtypes. As expected, our results clearly show that each SP subtype has a different pattern of associated factors; further, some factors only appear for one subtype and not for the others. Regarding animal fears, depression diagnosis, chronic diseases, blaming others, and threat control, while for the interference animal fears female gender seem to be critical phobia-specific factors. Although threat control appears for the Environmental subtype (fear) and female gender for the Situational subtype (interference). This is in line with previous studies showing gender differences in Animal phobia^47,51,52^. Further, a more restricted lifestyle and potential lack of social connections may also be associated with higher levels of fear and perceived interference^19,68^, which might also be the reason behind the slight overlap between Animal and environmental subtypes^69^. For the Environmental subtype, besides threat control, refocus on planning and depressive mood (fear), and self-blame were the unique factors. Environmental phobias comprise events and situations that can be foreseen and predicted and would not be possible to meet without the person approaching them. This might explain why one might blame oneself for the occurrence of an unfortunate event during a particular natural environment (e.g., water, heights) and highlights why planning and preparation can be a good coping mechanism. However, future studies are necessary to uncover the background mechanisms as Environmental phobias are understudied. We found that for Situational phobia fear was associated with traumatic experiences, while interference was associated with chronic diseases and worrying. This is in line with previous studies showing that excessive fear especially in this subtype is often evoked by one traumatic event in the past^70,71^. Then, the anticipation of an inevitable encounter with the object of the fear will trigger worrying, which in turn will impact the maintenance of the fear response, and increase the interference of the fear^72^. The unique predictor we found for BII phobia was marijuana consumption, and it was a protective factor. Similar to alcohol consumption we expected this to be a risk factor^19^, yet it seems that a recreational or at least subclinical form of alcohol and marijuana use may reduce fears. On the one hand, this might be a side effect, i.e., the consumption of these substances may reduce the reactivity of the individuals as shown in PTSD^73^, resulting in lesser fear and, thus, interference. On the other hand, people with these fears may be more likely to turn to these substances as self-imposed treatment, which temporarily could lower the level of fear and interference^74^. Finally, regarding the Other subtype, we found that stress control and worrying were the unique factors supporting previous studies on vomiting and choking-related fears^75,76^.

Limitations of the study include that we used a cross-sectional design, instead of gathering longitudinal data. Future studies are needed to test if the risk and protective factors suggested here would be predictive of the development of a SP. Further, the majority of our sample consisted mostly of females who are, according to our results and previous studies more prone to develop an SP. While this means the results are true for an endangered group of people, our results might not be universally true for other genders. Finally, although we included a large number of variables in the study, there could be more factors that can help predict the development of an SP or distinguish between subtypes. For instance, we targeted cognitive, but not behavioral or interpersonal ER strategies; we included measures of depression and anxiety but not tolerance of uncertainty or PTSD. Consequently, future studies are needed to explore all probable predictive variables.

In sum, these limitations notwithstanding, our study is among the first ones to explore phobia-specific patterns in the risk and protective factors that contribute to the development and maintenance of SPs. Our results may assist counselors, social workers, and other health professionals to identify individuals who might be at risk of developing an SP, and developing personalized treatment regimens. Younger females seem and people with a tendency to worry seem to be the most affected by such fears. Applications of our findings are also pertinent for both prevention and intervention strategies. Cognitive-behavioral-based interventions could be used to discourage the use of ER strategies—such as rumination, catastrophizing, and positive refocusing—that heighten fear levels, and instead focus on increasing the level of perceived control, and teach ER strategies—refocus on planning, in particular—that could lessen fears and its interference. These can be complemented by the inclusion of various phobia-specific factors. To further understand the socio-demographic, emotional, and personality-based mechanisms underlying the different phobia subtypes, future research should use longitudinal methods as well as experimental paradigms along with physiological measures.

### Supplementary Information

Supplementary Table 1.

Supplementary Table 2.

## Data Availability

The data that support the findings of this study are available from the corresponding author upon reasonable request.
